# Traumatic Tetanus Cured by Frictions with Tincture of Belladonna

**Published:** 1851-03

**Authors:** 


*Traumatic Tetanus cured by Frictions with Tincture of Belladonna.*—M. Bresse, surgeon at the Military Hospital at Rennes, proposed, in 1848, the treatment of traumatic tetanus by the application of tincture of belladonna, and reported a case, in which it had been successfully employed, in the "Gazette Medicale de Paris" of Sept. 30, 1848. M. Bresse has now placed on record another case which has come under his notice. The patient, one of the *Garde Mobile*, received a wound on the 20th of March; tetanic symptoms appeared on the 5th of April. Frictions of belladonna were commenced on the 6th, and by the 12th the patient was out of danger. Imprudently exposing himself to cold, the tetanic symptoms returned in the muscles of the back, but were quickly removed by again having recourse to the frictions. The tincture employed was composed of five parts of extract to eleven of alcohol, and was applied all over the body, and more particularly over the rigid parts.

M. Bresse adds, that another practitioner has arrested trismus, which he feared would proceed to general tetanus, by the same means.—*Gazette Medicale.—Med. Gaz.* from *Braithwaite's Retrospect.*

